# Enhancing Phenolic Contents and Antioxidant Potentials of *Antidesma thwaitesianum* by Supercritical Carbon Dioxide Extraction

**DOI:** 10.1155/2015/956298

**Published:** 2015-04-21

**Authors:** Warut Poontawee, Surapol Natakankitkul, Orawan Wongmekiat

**Affiliations:** ^1^Department of Pharmaceutical Sciences, Faculty of Pharmacy, Chiang Mai University, Chiang Mai 50200, Thailand; ^2^Department of Physiology, Faculty of Medicine, Chiang Mai University, Chiang Mai 50200, Thailand

## Abstract

Supercritical fluid extraction (SFE) has increasingly gained attention as an alternative technique for extraction of natural products without leaving toxic residues in extracts. *Antidesma thwaitesianum* Muell. Arg. (Phyllanthaceae), or ma mao, has been reported to exhibit antioxidant health benefits due to its phenolic constituents. To determine whether SFE technique could impact on phenolic contents and associated antioxidant potentials, ripe fruits of *Antidesma thwaitesianum* (Phyllanthaceae) were extracted using supercritical carbon dioxide (SC-CO_2_) and conventional solvents (ethanol, water). The results showed that the SC-CO_2_ extract contained significantly higher yield, total phenolic, flavonoid, and proanthocyanidin contents than those obtained from ethanol and water. It also demonstrated the greatest antioxidant activities as assessed by ABTS radical cation decolorization, DPPH radical scavenging, and ferric reducing antioxidant power (FRAP) assays. Further analysis using high-performance liquid chromatography with diode array and mass spectrometry detectors (HPLC-DAD/MSD) revealed the presence of catechin as a major phenolic compound of *Antidesma thwaitesianum* (Phyllanthaceae), with the maximum amount detected in the SC-CO_2_ extract. These data indicate that SFE technology improves both quantity and quality of *Antidesma thwaitesianum* fruit extract. The findings added more reliability of using this technique to produce high added value products from this medicinal plant.

## 1. Introduction

Trends in health promotion via antioxidant supplements have generated extensive demand in extracting biologically active substances from a variety of plant materials, primarily from polyphenolic-rich plants. Conventional solid-liquid extraction with organic solvents has commonly been operated; however, the production of undesirable residues during solvent removal has been reported and suggested as the cause of chronic toxicity of the final products [1]. According to the legal limitations of conventional solvents for food and pharmaceutical uses, supercritical fluid extraction (SFE) has been introduced as an alternative operation. This technology utilizes the extraction fluid at temperature and pressure above its critical value, where distinct liquid and gas phases do not exist. Carbon dioxide is the most frequently used solvent in supercritical extraction due to its low cost characteristics and being easily available in high purity form, nontoxic, noninflammable, and nonexplosive [2]. In addition, the solvent power of CO_2_ can be varied over a wide range by changing the operational conditions. Supercritical CO_2_ (SC-CO_2_) extraction has proved effective in the separation of desired compounds without leaving toxic residues in extracts. However, the commercial application of supercritical fluid technology remained restricted [1, 2].

Phenolic compounds are of particular interest for applications in the areas of functional foods and nutraceuticals due to their high antioxidant activities and abundance in the plant kingdom.* Antidesma thwaitesianum* Muell. Arg., or ma mao, is a native plant classified in the family Phyllanthaceae, genus* Antidesma*. It is commonly consumed as fresh fruit or beverages.* Antidesma thwaitesianum* has become an attractive fruit for production in scale of industry since recent studies have shown that it contains polyphenolic compounds, especially catechin and proanthocyanidins, and possesses antioxidant ability to protect human breast epithelial cells against oxidative damage [3–5]. However, the concern of organic solvent usage in these studies (i.e., methanol, chloroform, acetone, ethyl acetate, and hexane) hindered its advancement. The present study, therefore, investigated the feasibility of applying SC-CO_2_ extraction technology to overcome this drawback. The study outcomes may add more values to* Antidesma thwaitesianum* fruit, hasten its development as health supplements or pharmaceutical products, and promote the transfer of supercritical fluid technology to industry.

## 2. Materials and Methods

### 2.1. Chemicals and Reagents

All the chemicals and reagents used in this study were of analytical grade. Folin-Ciocalteu reagent was purchased from Merck, Darmstadt, Germany. Aluminum chloride hexahydrate (AlCl_3_·6H_2_O), ferrous sulfate heptahydrate (FeSO_4_·7H_2_O), and 2,2′-azino-bis(3-ethylbenzothiazoline-6-sulphonic acid) (ABTS) were obtained from Fluka (Darmstadt, Germany). All other chemicals were purchased from Sigma-Aldrich Co. (St Louis, MO, USA). Carbon dioxide with purity 99.5% was supplied by Lanna Industrial Gas. Co., Ltd. (Chiang Mai, Thailand).

### 2.2. Plant Materials and Sample Preparation

Ripe fruits of* Antidesma thwaitesianum* (harvested in August-September 2013) were obtained from Doi Kham Food Products Co., Ltd. (Royal Project, Chiang Mai, Thailand). The whole fruit was cleaned and washed under running tap water and rapidly stored at −20°C. The pulp was subsequently isolated, weighed, lyophilized, and ground into powder. The powdered sample was kept at 4°C in an air tight container protected from light until extracted.

### 2.3. Supercritical CO_2_ Extraction

Extraction was conducted using a laboratory scale supercritical fluid extractor unit (model SFE100, Thar Technologies Inc., PA, USA). The extraction protocol was set up according to the study of Yilmaz et al. [6] with slight modification. Briefly, 100 g of* Antidesma thwaitesianum* powder was loaded into the vessel and the supercritical extraction was carried out using the CO_2_ at a constant operating temperature of 40°C, pressure of 30 MPa, flow rate of 30 g/min, and the dynamic extraction time of 1 h. A 20 mL of 80% ethanol was used as cosolvent. The extract was collected in a preweighed collection glass vial and kept at −20°C ready for analysis.

### 2.4. Ethanol Extraction

*Antidesma thwaitesianum* powder was continuously shaken with 80% ethanol at room temperature for 4 h, centrifuged at 1000 ×g (Avanti 30, Beckman Coulter, CA, USA) for 15 min, and then supernatant was separated by filtration through Whatman No. 1 filter paper and rinsed with ethanol [7]. The extract obtained was ready for analysis.

### 2.5. Water Extraction

*Antidesma thwaitesianum* powder was extracted with distilled water for 4 h at room temperature. The extract was centrifuged at 1000 ×g for 15 min, filtered through Whatman No. 1 filter paper, rinsed with distilled water, and then used for analysis.

### 2.6. Calculation of Extraction Yield

The percent yield was calculated from the ratio between the total extract mass obtained (g) and the raw material mass loaded for extraction (g) on a dry weight basis. Each extraction procedure was done in triplicate, and the average of the three replicate was taken.

### 2.7. Determination of Total Phenolic Content

The total phenolic content was determined by the Folin-Ciocalteu method [8]. Briefly, appropriate dilutions of samples were oxidized with Folin-Ciocalteu reagent for 3 min. Then, the reaction was neutralized with 7.5% sodium carbonate solution and allowed to stand at 50°C for 15 min. The absorbance was measured at 760 nm and the total polyphenol content in each sample was calculated based on a standard curve, which was prepared using gallic acid and expressed as mg of gallic acid equivalent per 100 g of extract in dry weight (mg GAE/100 g dry wt).

### 2.8. Determination of Total Flavonoid Content

The aluminum chloride colorimetric method as described by Lin and Tang [9] was used to determine the total flavonoid content. Briefly, the reaction mixture containing the extracted solution, 95% ethanol, 10% AlCl_3_·6H_2_O, 1 M CH_3_COOK, was incubated at room temperature for 40 min. The absorbance was read at 415 nm and the flavonoid content was expressed as mg of quercetin equivalents per 100 g of extract in dry weight (mg QE/100 g dry wt).

### 2.9. Determination of Total Proanthocyanidin Content

A modified acid/butanol assay was used to quantify the total proanthocyanidin content [10]. Briefly, the assay mixture (extract solution, 77 mg of FeSO_4_·7H_2_O in 500 mL of HCl: *n*-butanol = 2 : 3) was incubated for 40 min at 95°C. The absorbance was read at 540 nm after cooling and results were expressed as mg of catechin equivalents per 100 g of extract in dry weight (mg CE/100 g dry wt).

### 2.10. Identification and Quantification of Phenolic Components

The extract solution of* Antidesma thwaitesianum* was centrifuged at 17000 ×g, 4°C for 60 min, and the supernatant obtained was directly analyzed by high-performance liquid chromatography with a diode array detector and a mass spectrometry detector (HPLC-DAD/MSD) using an HP 1100 liquid chromatograph equipped with MS detectors (Agilent Technology, CA, USA). Analyses were performed according to the modified method of Prasain et al. [11].

A C18 column 150 × 4.6 mm (5 *μ*m) (LichroCART, Merck, Germany) was maintained at 40°C, with a flow rate of 1 mL/min and detector fixed at 270 nm. The eluents were H_2_O acidified to pH 4.0 by 10 mM ammonium formate and formic acid (A) and acetonitrile (B). The following linear solvent gradient was applied: 100% A in 5 min, from 100% A to 80% A in 5 min, and a plateau of 10 min, to 60% A in 40 min. After separation, the HPLC effluent was delivered into a single quadrupole mass spectrometer (Agilent Technologies, USA) via orthogonal atmospheric pressure ionization-electrospray (API-ES) interface mode at 100–700* m/z* and step size at 0.2. The optimum electrospray ionization (ESI) conditions were as follows: ionization mode, positive (4,000 V), negative (3,500 V); nebulizer pressure, 60 psi; drying gas flow rate, 13 L/min; drying gas temperature, 320°C.

### 2.11. ABTS Assay

The ABTS^+^ decolorization assay was performed according to the method described by Luximon-Ramma et al. [12], based on the ability of an antioxidant to scavenge the preformed ABTS^+^ radicals relative to that of the standard trolox. Results were expressed in mg trolox equivalents/g of extract in dry weight (mg TE/g dry wt).

### 2.12. DPPH Assay

The DPPH radical scavenging assay was done according to the method of Brand-Williams et al. [13], based on the reduction of the stable radical, DPPH, to the formation of a nonradical form in the presence of hydrogen donating antioxidant. DPPH values were calculated using a linear regression equation of trolox standards, and the relative scavenging capacity of the extracts was expressed as mg trolox equivalent/g of extract in dry weight (mg TE/g dry wt).

### 2.13. FRAP Assay

The FRAP assay was performed according to Luximon-Ramma et al. [12]. The reaction mixture was read at 740 nm every 5 min in 1st h, every 10 min in 2nd h, and every 20 min in 3rd h. Results were expressed as mg trolox equivalents/g of extract in dry weight (mg TE/g dry wt).

### 2.14. Statistical Analysis

All the assays were performed in triplicate, and the results were expressed as mean ± SD from the three sets of observations. Comparisons were performed by one way ANOVA followed by Fisher's least significant difference (LSD) test using the SPSS 16.0 software (SPSS Inc., Chicago, IL, USA). *P* < 0.05 was considered statistically significant.

## 3. Results and Discussion

This research work examined the use of SC-CO_2_ extraction technology to extract* Antidesma thwaitesianum* fruits. The extraction yield, phytochemical contents, and antioxidant activity of the SC-CO_2_ extract were evaluated. The efficiency of the SC-CO_2_ extraction systems was compared to those of the conventional ethanol and water extraction methods. The results indicated that the best features of* Antidesma thwaitesianum* fruit extract were obtained by means of SC-CO_2_ extraction.

**Figure 1:**
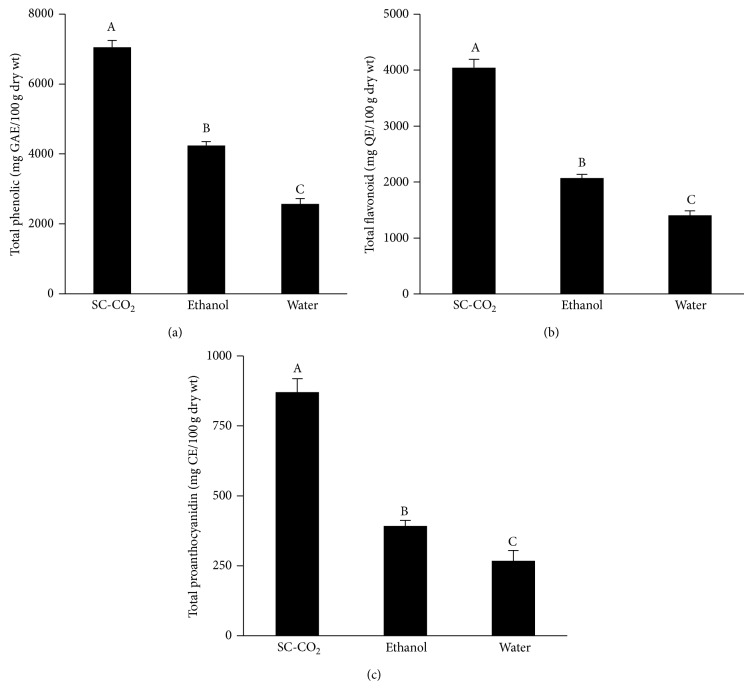
Effects of SC-CO_2_ extraction of* Antidesma thwaitesianum* on (a) total phenolic, (b) total flavonoid, and (c) total proanthocyanidin contents. Values are mean ± SD (*n* = 3). GAE, gallic acid equivalent; QE, quercetin equivalent; CE, catechin equivalent. Means with different letters (A–C) are significantly different (*P* < 0.05).

In the present study, supercritical fluid extraction, a clean technology, was operated using CO_2_ as an extraction solvent. It is observed that extraction using SC-CO_2_ gains more product yield about 1.5-fold than that of ethanol (17.87 ± 0.55 and 11.50 ± 1.08%, resp., *P* < 0.05) and almost 3 times higher (*P* < 0.05) in the case of water (7.67 ± 0.87%). The extract from SC-CO_2_ also exhibited the highest contents of total phenolic, total flavonoid, and total proanthocyanidin, about 2-3-fold higher (all *P* < 0.05) when compared with the other extracting solvents (Figure 1). Previous studies have shown that the levels of yield as well as phenolic compounds can be altered by processing conditions and the extracting solvent [14]. CO_2_ at supercritical conditions can effuse through solids like a gas and dissolve materials like a liquid; thus, it has a higher diffusivity as well as lower density, viscosity, and surface tension compared to ethanol and water [15]. In this regard, the favorable transport properties of SC-CO_2_ allow deeper penetration into solid plant matrix and more efficient and faster extraction than conventional organic solvents, causing higher extraction yield and phytochemical contents as observed in this study. The rise in extraction yield by SC-CO_2_ may also be the result of interaction between extraction temperature and pressure. This suggestion is based on evidence showing that both factors can increase the extraction yield by increasing the solvent power and selectivity of SC-CO_2_ [16]. Although raising operation pressure and temperature could possibly gain more yield, the extraction process in this study was set at constant temperature of 40°C and pressure of 30 MPa, as this condition is considered suitable for thermolabile compound to avoid degradation [6].

Another reason for getting the higher amounts of yield and polyphenols by SC-CO_2_ extraction in the present experiment, particularly over that of water extraction, may lie in the use of ethanol as cosolvent. Because CO_2_ is nonpolar and has some limitation for extracting of polar compounds, the use of cosolvents to overcome this drawback is required [15]. Ethanol has widely been used for this purpose because water is not as miscible with CO_2_ as alcohol, while methanol is environmentally hazardous and toxic to human health. Several lines of evidence have indicated that ethanol can enhance extraction yield by increasing the solubility of polar solutes [17], inducing changes in the structure of cellular matrix via intracrystalline and osmotic swelling [18], and breaking analyzed matrix binding by competing with polar interaction between matrix and compounds to be extracted [16]. In addition, increasing the amount of ethanol in the supercritical fluid has been shown to decrease the extraction time and the consumption of CO_2_ [19].

**Figure 2:**
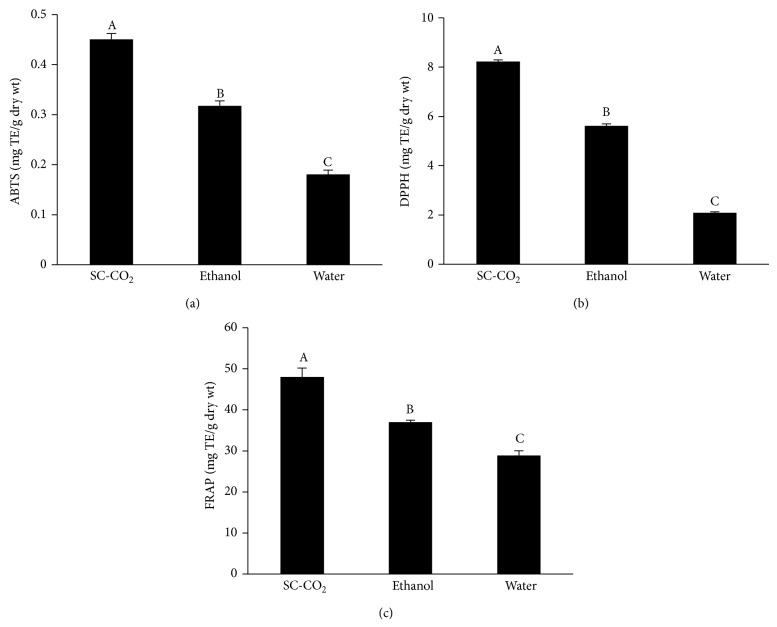
Effects of SC-CO_2_ extraction on antioxidant activity of* Antidesma thwaitesianum* as assessed by (a) ABTS, (b) DPPH, and (c) FRAP. Values are mean ± SD (*n* = 3). TE, trolox equivalent. Means with different letters (A–C) are significantly different (*P* < 0.05).

Because of the complex nature of polyphenols, the antioxidant activities of plant extracts cannot be evaluated by only a single method. In this study, the ABTS, DPPH, and FRAP assays were used to determine the antioxidant potentials of* Antidesma thwaitesianum* extracts and the results are shown in Figure 2. The data clearly demonstrated the significantly greater (*P* < 0.05) scavenging activities of the extract from SC-CO_2_ against ABTS and DPPH radicals than those of the ethanol and water. Ferric reducing antioxidant power also showed similar profile as the radical scavenging activities in which the greatest ability (*P* < 0.05) was detectable from the SC-CO_2_ extract.

**Figure 3:**
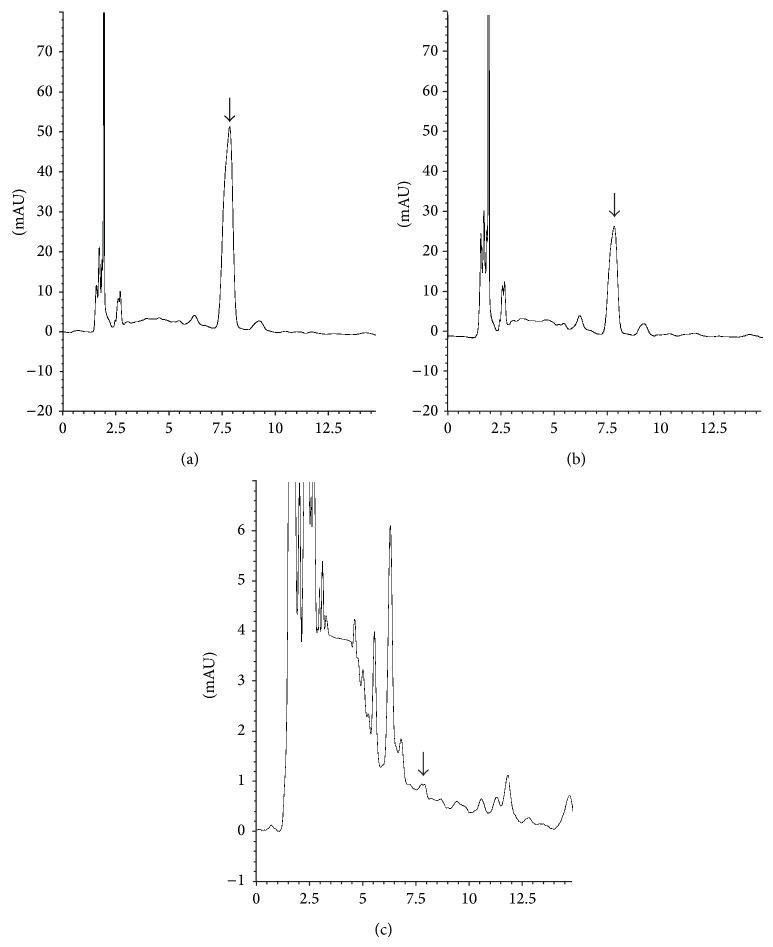
Chromatogram at 270 nm of the extract from* Antidesma thwaitesianum* by (a) supercritical carbon dioxide, (b) ethanol, and (c) water extraction. Arrow indicates catechin peak at retention time 7.84 min.

**Figure 4:**
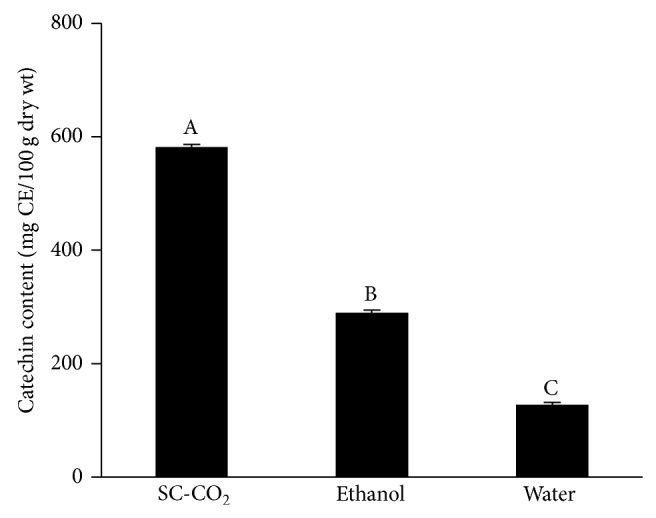
Effects of SC-CO_2_ extraction on catechin content of* Antidesma thwaitesianum*. Values are mean ± SD (*n* = 3). CE, catechin equivalent. Means with different letters (A–C) are significantly different (*P* < 0.05).

HPLC-DAD/MS assay was used to further characterize the phenolic compounds responsible for the antioxidant activity of* Antidesma thwaitesianum* fruit extract. However, the main focus of this study was on catechins as they are ubiquitous constituents of vascular plants and frequent components of traditional herbal remedies. Its antioxidant activity is also well established [20]. Most importantly, catechin has previously been reported to be the major phenolic compound in the extracts of* Antidesma thwaitesianum* by conventional solvent extraction methods [4]. Using catechin as a standard reference, the content of catechin identified in the SC-CO_2_ extract was approximately 2 times and almost 5 times higher (*P* < 0.05) than that recorded from the ethanol and water extracts, respectively (Figures 3 and 4).

In general, the radical scavenging property and antioxidant activity of plant extracts are positively correlated with their phytochemical contents. Applying this concept to the present results, it is reasonable to conclude that the greatest extraction yield as well as the highest amounts of phytochemicals (phenolics, flavonoids, and proanthocyanidins, primarily catechin) is responsible for the greatest antioxidant potentials of the SC-CO_2_ product. In addition, previous study demonstrated that the extract obtained by conventional organic solvent extraction can undergo oxidative transformations during solvent removal [3]. From this viewpoint, supercritical extraction uses pure gas and the CO_2_ processing creates a medium without oxygen where oxidation reactions can be avoided, thus allows the recovery of the extract with high purity, completely free of solvent, and retains their antioxidant potency.

## 4. Conclusions

The current investigation demonstrates that both quantity and quality of the* Antidesma thwaitesianum* fruit extract are improved by supercritical CO_2_ extraction. These findings underscore the efficiencies of supercritical technology over traditional solvent extraction methods. Due to the legal restrictions in food technologies and the increasing demands for safe and powerful natural antioxidants, it is suggested that supercritical CO_2_ extraction is a promising technology for extraction and purification of antioxidant compounds from plant materials including* Antidesma thwaitesianum*.
